# Observations Onn Medical Reform

**Published:** 1806-06

**Authors:** 


### *To the Editors of the Medical and Phyfical Journal.*

GENTLEMEN,

THE note attached to my communication of Feb. 11, would have prevented me troubling you any farther on the subject of Medical Reform, had I not observed some remarks on my paper by Dr. Cayley of Ripon, in the Medical Review of the last month. By this gentleman, I am called upon to come forward in my real character; and as I have chosen to remain in the shade, he undertakes to throw light upon me, and by the aid of his powers of illumination, discovers that I either do not belong to the profession, or practice irregularly. All this an anonymous writer must bear with, and I am well contented to submit, provided you allow my reasons for still continuing undiscovered. I am situated near the focus of medical reform, and am thereby enabled to form opinions on the motives of some of the principal performers in this plan; which persons at a distance might pass over unobserved. I know too much of human nature to suppose, that an individual can oppose himself successfully to the prejudices of a considerable number of persons, or to believe that the majority will suffer their opinions to be commented upon with impunity; impunity; I therefore think my probability of being useful depends upon my remaining concealed. Happy shall I be if my warning voice calls the profession of medicine to a consideration of its true interest, before it has so far committed itself as to be saddled with a tax never to be removed. For, whether or not I profess regularly or irregularly the practice of medicine, I feel the highest esteem for the numerous members of the profession, who spend their labor, time, and constitution in the exercise of medical avocations. Thus much as to myself and my views, which I am really ashamed to have obtruded on public notice.

Now let me turn your observation to the subject of medical reform, the objects of the reformers, and the means by which they promise to attain their end.

Dr. Harrison has published a small-part of his correspondence in the Medical Review; and if we may judge by this specimen of the whole, we may reasonably fear the learned Doctor will have more difficulty in reconciling the jarring opinions of his various correspondents, than he had formerly in forming a plausible theory of the cause, and a rational method of cure of the rot in sheep, from the answers to his queries on that subject. It is strikingly evident that all his correspondents reason only from their own particular experience, and adopt the system of reform only as it may ameliorate their own situation. Thus the surgeon apothecary hopes, by bribing government in the payment for his licence, to shut out the druggist from the composition of medicine; while the physician, by the same expedient, means to chain the apothecary to his counter, never to presume beyond the mingling ingredients in a mortar. If such is to be the occupation of the surgeon apothecary, surely no complaint need be made of the deficiency of the present medical education; as no one will contend for a very profound knowledge of languages being necessary to the cook-maid, who compounds the necessary ingredients for our support; and with little instruction would prepare the receipts necessary to restore health. For in what does the dispensatory differ from the manual of Mrs. Glasse, except that in the one the receipts are simple and clothed in a learned language, while in the other they are complex, but conveyed in the vulgar idiom. But the great mass of the people, on whom disease most frequently falls, and where its progress is most to be dreaded, are unable to defray the expence incident to the attendance of a physician, and physicians are equally unable to give up their time without an adequate compensation; there must therefore be some intermediate person, and such custom has prescribed in the surgeon apothecary; who is contented to give his time and medicine for a trifling addition to the real cost of the medical article administered; an advantage perfectly consistent with all our principles of right, as is proved by the ingenious Dr. Adam Smith in his Wealth of Nations.

As, therefore, the profession of Medicine possesses no advantage beyond other occupations, why are the professors of it to be taxed with an imposition not levied upon their fellow subjects? I shall be answered, the profession of the Law already submits to a fine on the admission of its practitioners, and the respectability of the body has much amended by it; this is allowed, but the courts are shut against all unqualified persons, and therefore the lawyer is amply repaid his fine, the competitors in the field being fewer, and his practice proportionably increased. Could this obtain in medicine? Certainly not; for what penal statutes can prevent any individual from submitting his health to the direction of a weaver, a blacksmith, or a farrier? or what means can be devised to root out from the human mind, the love of mystery, or prevent such characters from boasting and profiting by their nostrums?

But it is said, the proposed reform is to establish a just distinction between regular and irregular practitioners; that all such as submit to its constitutions are to be considered as regular practitioners, will have the privilege of paying an annual sum to government, and appear in the public papers once or more times in a year, as being licensed to use those instruments of death, called pills and potions, in the same method, as persons taking a game licence now appear; while those who refuse to submit to these conditions, will keep their money, and be considered as mere poachers in the field of death; but, like their brethren in the pursuit of game, will frequently profit more by their pursuits than the qualified sportsman. If additional expense, or additional honours had any influence with the public, the physician practicing under a degree from an English University, should be preferred to him who bears a Scotch diploma; the Member of the College of Surgeons should be called in preference to the surgeon-apothecary practising without any approved testimonial of his competency. But facts, I am sorry to observe, are strongly against any such preference; in many instances the graduate of Edinbro', or even St. Andrew's, rolls in his carriage, age, while his humble competitor from an English University is contented to walk the streets in quest of his daily fees. And much I fear that many members of the Royal College of Surgeons regret their folly in parting with solid pelf for the honorary but useless pageant of a diploma.

This being the fact, as to the public bodies at present authorised to judge of medical knowledge; can we hope for any better effect from the crude system of medical reform, now presented for our approval?

The College of Surgeons is authorised to confer diplomas, and receives a handsome compensation from the candidate for the honour conferred; in the same manner that the licentiate of the College of Physicians is charged with a considerable sum for his licence. Both these persons might reasonably consider themselves, as under the protection of the law, in the exclusive exercise of their profession; but we do not find, even in cases of medical jurisprudence, that any stress is laid upon the medical person, called in evidence, having a degree from either of these authorities; nor has it ever been known, that a judge has reprimanded a person assuming a medical character without such authority; or that, as an expounder of the law, he has ever pressed it upon the public, that these two colleges were the only authorised approvers of medical science. Even cases are not wanting, where public offices of medical trust have been conferred on persons bearing no diploma from either college.

Dr. Cayley has taken particular offence at the expression, the fallacy and iniquity of medical reform; but I will ask the Doctor if that system is not fallacious, which undertakes to remedy, what cannot be restrained by human laws, namely, opinion? And whether it is not an act of iniquity to charge persons with an annual expence under the promise of a benefit, impossible to be secured to them? Is the situation of medical men so prosperous, as to enable them to bear additional burthens? Do they not in common with their fellow subjects bear the still increasing burthen on income? and the example of some of the leading reformers shews how grievous this burthen is to bear. Are they not taxed for their servants and horses, the implements of their profession? Do not the lower practitioners pay largely for the assistance necessary in their shops, whether as assistants or apprentices, on whom also government receives a tax? Add to these public burthens the difficulty of recovering medical fees, and the ill will with which medical assistance is repaid; and it will be no falsity to assert, that the faculty of medicine already bears more than an equal share of taxation. With these considerations in view, let me beg your numerous readers to pause before they rashly assent to a scheme which will assuredly end in taxation; while all the benefits to be derived to the profession in general will be frittered away in trifling distinctions. Such distinctions may be gratifying to the first movers of medical reform, by elevating them above their usual sphere of action, but will afford little satisfaction to the industrious and conscientious practitioner, who will find his annual contribution in no way to contribute to his reputation or emolument.

